# Exposure of infants to organochlorine pesticides from breast milk consumption in southwestern Ethiopia

**DOI:** 10.1038/s41598-021-01656-x

**Published:** 2021-11-11

**Authors:** Seblework Mekonen, Argaw Ambelu, Mekitie Wondafrash, Patrick Kolsteren, Pieter Spanoghe

**Affiliations:** 1 Department of Environmental Health Sciences and Technology, Jimma University, P.O. Box 378, Jimma, Ethiopia; 2 St. Paul’s Hospital Millennium Medical Collge, Addis Ababa, Ethiopia; 3 Department of Food Technology, Safety and Health, Faculty of Bioscience Engineering, Ghent University Coupure Links 653, 9000 Ghent, Belgium; 4 Department of Plants and Crops, Faculty of Bioscience Engineering, Ghent University, Coupure Links 653, 9000 Ghent, Belgium

**Keywords:** Environmental social sciences, Biomarkers, Risk factors

## Abstract

Breast milk is often used as an environmental bioindicator since it serves as an important medium to accumulate organochlorine pesticides. The main aim of this study is to determine the level of organochlorine pesticides in human breast milk collected from three districts of southwestern Ethiopia (Asendabo, Deneba, and Serbo) at three times points baselines (1st month), midline ( six months), and end line (12 months) and risk of infants’ exposure. A longitudinal study was conducted to assess pesticide residues in human breast milk samples and evaluate the risk-exposure of infants to these pesticides from consumption of mother’s milk in Ethiopia. Breast milk samples were collected from 168 mothers at three time points and pesticides were extracted using the quick, easy, cheap, effective, rugged, and safe (QuEChERS) method. The level of pesticide residues in human milk samples and exposure of infants to the pesticides was evaluated by calculating the estimated daily intake and compared with the provisional tolerable daily intake of the pesticides under study. The results indicated that, from the eight organochlorine pesticides analyzed in 447 breast milk samples at three sampling times, DDT and its metabolites were detected. p,p′-DDE and p,p′-DDT were detected in all (100%) of the breast milk samples while, p,p-DDD, and o,p-DDT were detected in 53.9%, and 42.7%, respectively. The mean concentration of total DDT at three time points(baseline, midline and endline) were 2.25, 1.68 and 1.32 µg/g milk fat, respectively. The mean concentration of total DDT from the three districts was 1.85 µg/g milk fat which is above the maximum residue limit (MRL = 0.02 µg/g milk fat set by FAO/WHO). The mean ratio of DDT/DDE for the three areas was calculated less than five (< 5) indicates historical DDT use in the study area. The estimated daily intake of infants at the first month of breastfeeding was 11.24 µg/kg-BW/day, above the provisional tolerable daily intake (PTDI) for total DDT set by FAO/WHO, which is 10 µg/kg body weight. An intake of OCPs is a big concern for infants' health in Ethiopia and countries with a similar condition, particularly at the first month of lactation. Strict regulations of the health-threatening pesticide by the regulatory body (Environment, Forest and Climate Change Commission) at the country and regional levels is advocated.

## Introduction

Pesticides are often promoted as input to increase agricultural productivity by decreasing pre or post-harvest losses. In contrast, the production and intensive use of pesticides led to widespread contamination of the environment to the level it might impact human health^1^. However, organochlorine pesticides (OCPs), such as dichlorodiphenyltrichloroethane (DDT), aldrin, dieldrin, heptachlor, heptachlor-epoxide, lindane, and other similar agrochemicals have been used worldwide, for several decades. As this wide intensive use triggered environmental, before 15 years, Ethiopia declared it as one of a national problem^2^. OCPs are characterised by bio-accumulative nature due to lipophilic properties, especially in the food chain, where they find their way into the human body. Although many developed countries banned these products years ago, several African countries still use OCPs, especially for the prevention and control of malaria^3,4^. According to the Stockholm convention, OCPs are considered dangerous due to their persistency, bioaccumulation, toxicity, and long-distance pollutant transport characteristics^5^. Following this convention, the Ethiopian government stopped use of DDT for Agricultural purpose but for malaria control^6^. However, DDT application was stopped following the development of resistance to DDT among the malaria disease vectors^7^.

Their long-term uses and persistent nature bring them as ambiguous contaminants in the environment and cause risks to human health. For Example, the detection of DDT and its metabolites DDE has a significant impact on human health. They are regarded as endocrine disrupters, probable carcinogens, and cause teratogenicity to the future generation^8^.

The exposure of living organisms to OCPs compounds in our ecosystem depends on their position or trophic levels in the food chain^9^. OCPs contaminated foods such as fruit, vegetables, cereals, and dairy products are considered the primary source of pesticides to the human body^10^. Exposure of humans to organochlorine pesticides results in accumulating the OCPs in the fat tissue^11^. In Ethiopia, breast milk is the commonly consumed food item for infants, especially until six months from their birth date. Ministry of Health in Ethiopia insists exclusive breast milk feeding until six months of age of the infant. Children's diets are one of the routes of exposure to environmental chemicals. Exposure through diet is critically important as children consume more food relative to their body weight than adults. Their foods are less varied, and this allows for a greater opportunity for continuous exposure to environmental contaminants, including organochlorine pesticides^12^.

Breast milk with relatively high-fat content is considered a suitable matrix for highly bio-accumulative chlorinated pesticides. It can be used as the best indicator to study the long-term human health impacts of these chemicals^13,14^. Breast milk is easy to collect non-invasively and important to indicates the contaminant levels in maternal fat^15^. Additionally, monitoring of breast milk for the determination of OCPs provides a means of estimating the intake by breastfeeding infants^14^. The exposure of women during pregnancy to these OCPs may lead to various health problems for fetus such as low birth weight, disturbance of thyroid hormone, and neurodevelopmental delay^16^. In malaria-endemic areas, DDT level in breast milk usually exceeds the maximum residues limits, and its intake by infants significantly exceeds the Provisional Tolerable Daily Intake (PTDI)^17,18^. Of particular concern, breastfeeding women living in malaria-endemic areas and indoor residual spray (IRS) treated houses can transfer OCPs to their infants via lactation^3^. Critical attention is and intervention is needed for the presence of these highly bioaccumulative chlorinated pesticides in foods designed for small children^19^.

Studies^3,14^ have detected OCPs in human breast milk. Studies done in Asian countries documented DDT from breast milk at a high concentration (3.2 µg/g fat) in India^20^ and a low concentration (0.019 µg/g fat) in Taiwan^21^.

Being an agrarian country, Ethiopia is among the primary users of OCPs for increasing agricultural yields. Besides, organochlorine pesticides such as DDT were highly used in Ethiopia for the control of vector-borne diseases^22^.. The large-scale use of OCPs has resulted in significant exposure to the local community, particularly to high-risk groups such as infants. The study conducted by Gebremichael and colleagues^23^ on the residues of organochlorine pesticides in human breast milk attracted our attention to undertaking a detailed investigation during the one year lactation period. Our previous study from the Jimma zone, where this study is conducted, indicated that mothers could be highly exposed to OCPs from drinking water and staple food items they are consuming^24^. Therefore, the main aim of this study is to determine the level of organochlorine pesticides in the breast milk of mothers living in southwest Ethiopia and potential risks to infants in Ethiopia.

## Methods

### Study area, design, and period

The study was conducted in three districts of the Jimma zone, namely Serbo, Asendabo, and Deneba in southwestern Ethiopia. The area lies in a midland agro-climatic zone where subsistence farming is the predominant form of livelihood. The three districts are malaria endemic. From a study done by Mekonen and colleagues^25^, in Jimma zone where the three districts are located, infants consuming maize as complimentary food were reportedly exposed to a high level of total DDT. Breast milk samples were collected during the Randomized Control Trial (RCT) study for Omeg3 from November 2013 to February 2015^26,27^. In 2018–2019 the breast milk samples were further analyzed to quantify the amount and presence of organochlorine pesticides.

According to Argaw and his colleagues^26,27^, study participants were identified from the Gilgel Gibe Health and Demographic Surveillance System (GGHDSS). The breastfeeding mothers with singleton infants and with no intention to leave the study area for more than one month over the coming year were included. Mother-infant pairs with known chronic illness or taking other nutritional supplements; infants with a congenital abnormality that could affect breastfeeding or an infant having severe anemia (hemoglobin < 7.0 g/dl) were excluded from the study. Hence, a subsample of 168 mother-infant pairs were identified to provide breast milk samples at baseline (at 1st month), midline (6th month), and end line (12th month). However, not all breast milk samples were available from the three time points for pesticide residue analysis. 447 breast milk samples in three-time points were analyzed for DDT and its metabolites (p,p-DDE, p,p-DDD, o,p-DDT and p,p-DDT), aldrin, dieldrin, endosulfan α, and hexachlorobenzene. The highest number of samples analyzed were from Asendabo, followed by Deneba, and then Serbo were 256, 106, and 85, respectively.

On top of the breast milk samples, every mother completed a questionnaire about personal information such as maternal age, maternal weight and height, educational background, number of children, and place of residence.

### Breast milk sample collection and storage

After the mother was allowed to breastfed her child for few minutes, a trained female clinical nurse expressed a total of 447 breast milk samples (~ 10 ml each) manually or using breast pumps as needed into a sterile plastic container with lids. It was labeled and kept in a cool bag at − 20 °C while it was transported from the field. On the same day of breastmilk collection, 9 ml of homogenized milk sample was pipetted into a 10 ml screw-capped cryovial containing 1 ml BHT (2, 6-Di-tert-butyl-4-methylphenol) solution and was then stored at − 80 °C in a laboratory in Jimma University. Finally, the sample was shipped in dry ice to a laboratory in Ghent University (Belgium) and stored at − 80 °C till extraction and instrumental analysis^26^. The breast milk samples were collected from the laboratory in the Department of Food Safety and Quality, Faculty of Bioscience Engineering, Ghent University and were brought to the laboratory of Crop Protection Chemistry, Faculty of Bioscience Engineering, Ghent University Coupure Links 653, 9000 Ghent, Belgium, for the extraction and determination of the presence of organochlorine pesticides under study.

### Materials and reagents

The materials and reagents used to analyze organochlorine pesticides in human breast milk were similar to the previously published work of Mekonen, et al.^24^. Analytical grade reagents, solvents and extraction techniques were used.

### Method validation

To evaluate the validity of the analytical procedures during laboratory analysis, method validations, such as determining the limit of detection, the limit of quantification, repeatability (%RSD), and % recovery, were undertaken. As was difficult to get blank breast milk, the validation test was done using baby formula milk as a blank sample. A standard working solution, 100 µL of 1 mg/l was spiked in 10 ml of formula milk for each pesticide under study. Then, the samples were extracted using the quick, easy, cheap, effective, rugged, and safe (QuEChERS) method. The determination of pesticides was done using Gas Chromatography with an Electron Capture Detector (GC-ECD). The extract was cleaned by dispersive solid-phase extraction (d-SPE) using Magnesium Sulphate (MgSo4), C18 (Octadecyl) and Primary Secondary Amine (PSA) as an adsorbent. The linearity of the chromatographic analysis was assessed by running an external standard solution at a concentration range of 0.004–0.1 mg/l and the calibration line is drawn with a determination coefficient > 0.99 for all the pesticides under study. The concentration of the OCPs under this study was quantified based on the regression equitation obtained from the standard curves and by comparing the peak areas. The recovery studies were done by spiking a mixture of eight OCPs at a concentration of 0.01 ppm with seven replicates. The %recoveries were calculated by dividing the average concentration observed by the spiked concentration multiplied by one hundred (100) and was in the acceptable analytical range (70–120%) for all the pesticides.

### Extraction and clean up

All sample preparation and experimental monitoring were undertaken in the laboratory of Crop Protection Chemistry at Ghent University, Belgium. Extraction and cleanup of the breast milk samples were conducted using the QuEChERS extraction followed by dispersed solid-phase extraction (dSPE) cleanup method, as recommended by the AOAC official method 2007.01^29^, for fatty substances with slight modification. The milk samples were shaken first to make it homogenous and 2 mL of subsamples were taken in 50 mL polypropylene centrifuge tubes and 8 ml of reagent water was added. Then 10 mL of acetonitrile as an extraction solvent was added and the mixture was allowed to stand at room temperature for 30 min and shaken by hand every 10 min. All other procedures have followed the methods of the previously published paper in our research group^24^. The concentration of organochlorine pesticides was calculated per gram of fat in human breast milk^30^, stated from the composition of breast milk, the fat content is 3.5 g of fat per 100 ml of breast milk.

### Estimated daily intake calculation

Breastfeeding is an elimination process for the mother, and the body burden of pesticide residues will be decreased accordingly. But for infants, it is a major route of exposure to pesticides, and therefore, the health risk of infants exposed to pesticides should be evaluated carefully. For the present study, the estimated daily intakes by infants were calculated by assuming similar consumption in developing countries. The breast milk consumption data and the specific gravity of breast milk (1.030 kg/l) was taken from a study done by^31^ at each age groups (1st, 6th and 12 months of lactation) and according to^30^, from the composition of breast milk, the fat content is 3.5% of fat per 100 ml (3.5 g/100 ml) of breast milk. The estimated daily intake of infants was calculated based on the formula for the calculation of estimated daily intake was according to^32^. The body weight of infants (BWinfant) were measured during breastmilk sample collection.

$${\text{EDI}}_{{{\text{pesticides}}}} = {\text{Cmilk}}\;(\upmu {\text{g/g}}) \times {\text{C}}_{{{\text{fat}}}} \times {\text{M}}_{{{\text{ingestion}}}} ({\text{g/day}})/{\text{BW}}_{{{\text{infants}}}} \;({\text{kg}})$$

where EDI is the estimated daily intake of infants (μg/kg BW/day), Cmilk is the concentration of pesticides in milk (μg/g fat), Cfat is the lipid content in milk (%), M_ingestion_ is breast milk intake of infants (g/day); BWinfants is the bodyweight of infants (kg).

**Figure 1:**
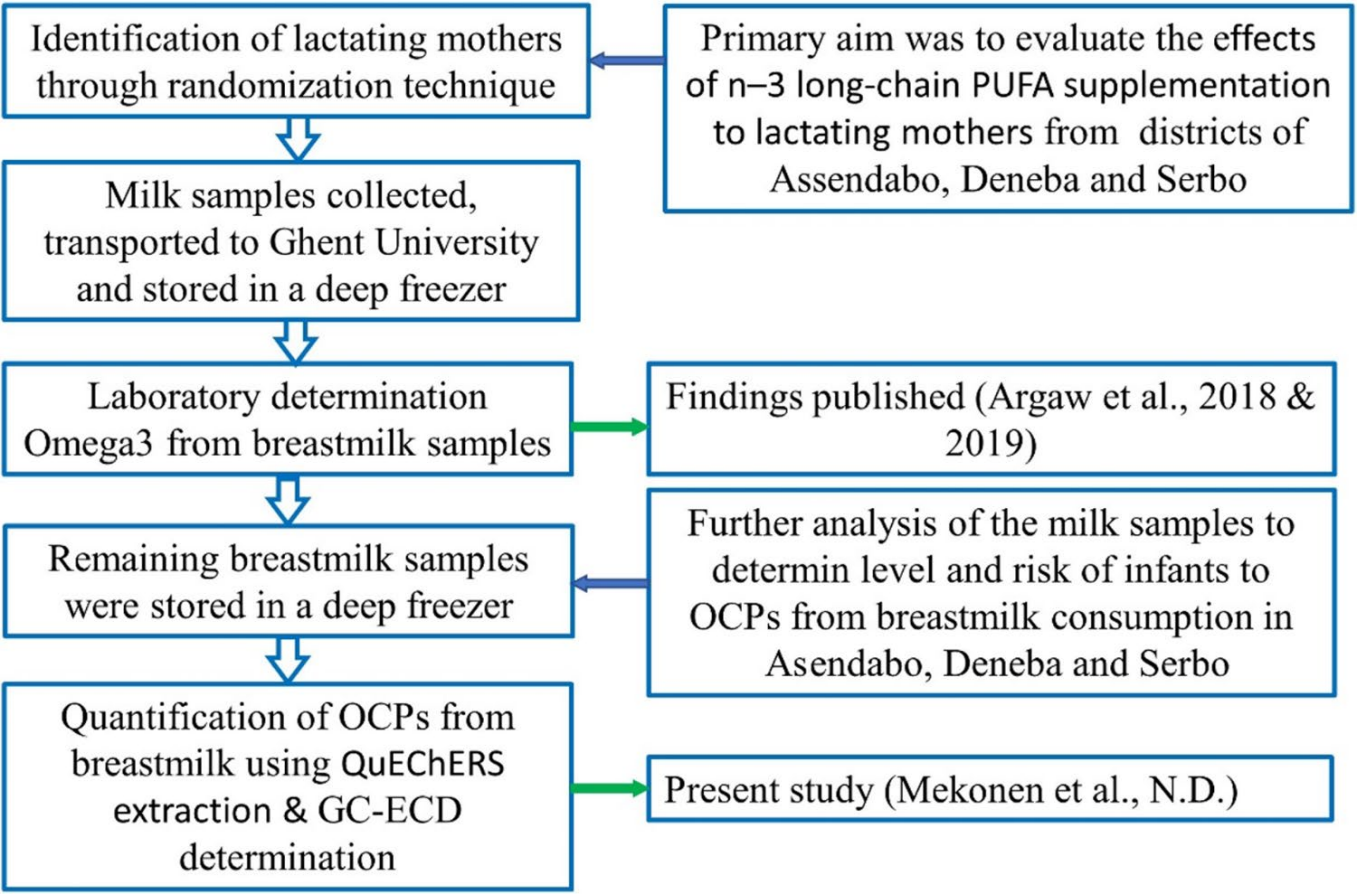
Flow diagram of the study showing general steps from sample source to OCPs residue data generation.

The flow diagram of the general study is described in Fig. 1.

### Instrument analysis of the samples

Identification, separation, and quantification of organochlorine pesticides in human breast milk were undertaken using gas-chromatography with an electron capture detector (GC-ECD, Agilent Technologies 6890 N) mounted with an autosampler. HP-5 capillary column of 30 m × 0.25 mm i.d. × 0.25 μm film thickness coated with 5% phenyl methyl siloxane (Model number Agilent 19091 J-433) in combination with the following oven temperature program. The initial temperature was 80 °C, ramp at 30 °C min^-1^ to 180 °C, ramp at 3 °C min^-1^ to 205 °C, held for 4 min, ramp at 20 °C min^-1^ to 290 °C, held for 8 min, ramp at 50 °C min^-1^ to 325 °C. For deltamethrin determination, the oven temperature was maintained initially at 130°c, held for 1 min, ramp at 30 °C min^-1^ to 280 °C, held for 16 min and ramp at 50 °C min^-1^ to 325 °C, held for 3 min. The total GC run time was 27.92 min. Helium (99.999% purity) was used as a carrier gas at a flow rate of 20 mL min^-1^ and nitrogen as a makeup gas at a flow rate of 60 mL min^-1^. An aliquot of 1µL was injected in split mode at a Split ratio of 50:1 and an injection temperature of 280 °C. The pesticide residues were detected with Electron Capture Detector (µ-ECD) operated at a temperature of 300 °C.^24^.

### Quality control

All methods were performed in accordance with the relevant guidelines and regulations.

The sample analysis was performed in replicate. A solvent blank was run before samples were analyzed every day. All measurements were performed within the range of linearity. OCP determination was done on the same day after calibration standards were run to obtain consistent results. The trueness or accuracy was determined by processing the spiked baby formula milk and the % recovery was calculated and obtained in the acceptable analytical range. The reproducibility of the analytical method was checked by spiking a known concentration of the pesticide standard at seven replicates, followed by the determination of %RSD. The limit of detection (LOD) and limit of quantification (LOQ) were determined in analytical blanks from procedural values (LOD = 3 times the standard deviation of the replicates) and limit of quantification (LOQ = 10 times the standard deviation of the replicates). The linearity of the chromatographic analysis was done by running five different concentrations from 0.004–01 mg/l and the coefficient of determination was calculated to be > 0.99. The mean levels of the undetected pesticide residues in the breast milk sample were calculated with the assumption of zero (ND = 0)^20^ for the ease of statistical analysis.

### Statistical analysis

Data analysis was performed using the R statistical package^33^. The concentration of organochlorine pesticides in human breast milk was summarized using descriptive statistics such as mean, median, standard deviation, minimum and maximum values. A multiple linear regression model was used to identify the predicting variables to a concentration of total DDT in breast milk. After the normality of the dependent variable was checked, log transformation was made where the skewness of the data was improved from 4 to 0.83. Mulitvariate analysis of variance (MANOVA) was performed to investigate the presence of concentration difference at the three stages of breastfeeding. In addition, Repeated Measure ANOVA and post hoc tests were used to compare DDT concentrations of breastmilk samples at different stages (times) and between other categorical variables. In addition, generalized linear mixed model was fitted to identify the predicting variables using ‘lme4’ library in R statistical package. A p < 0.05 was considered to be statistically significant.

### Ethical approval and consent to participate

Data and milk samples were collected after ethical clearance was issued by the Institutional Review Board of Jimma University in Ethiopia, the Ethics Committee of Ghent University Hospital in Belgium, and the National Health Research Ethics Committee of Ethiopia approved the study protocol (Registration Number: B670201214299). Besides, each study participant was included in the study after verbal and written consent is obtained.

## Results and discussion

### Method validation results

Except for endosulfan α (52.5%), the % recovery of the pesticides under study ranged from 71.1% for p,p-DDE to 117.5% for Aldrin. This was in the acceptable analytical range (70–120%) and indicated all the OCPs under study were well recovered by using the QuEChERS method. The percent relative standard deviation (%RSD) ranged from 1.61 to 12.81%, which indicates that the method is repeatable (%RSD < 20). The chlorinated pesticide endosulfan α is less recovered (< 60%) while the percent standard deviation shows repeatability (%RSD < 10). From a study done by^34^, the modified QuEChERS method is known to obtain lower recoveries for lipid-soluble pesticides such as endosulfan extracted from fatty matrices. The limit of detection (LOD) and limit of qualifications (LOQ) expressed per gram milk fat bases were 0.018–0.078 µg/g milk fat and 0.062 to 2.38 µg/g milk fat, respectively. The linearity of the chromatographic analysis was done by running five different concentrations and the coefficient of determination(r^2^) calculated was > 0.99 for all the pesticides under study. From these results, the QuEChERS extraction is a valid method of determining organochlorine pesticides in breast milk.

### General demographic characteristics of the study participants

Of the mothers who participated in the study, more than 75.9% lived in the three districts' towns (Asendabo, Deneba and Serbo). In contrast, while the others came from the rural areas of the Jimma zone southwest, Ethiopia. The median age of the mothers was 25 years where the minimum is 18 and the maximum is 45 years. Regarding the educational status, 81 (48.8%) mothers had no formal education, 57 (34.3%) followed primary education (grade 1–8), and 28 (16.9%) followed secondary (grade 9th and above) school. This indicates that most of the participating mothers were not educated and this can be one factor to increase the exposure to pesticides, perhaps educated individuals will take necessary precautions from being exposed to contaminants^35^. The mean body weight of infants at 1st month (baseline), 6th month (midline), and 12 months (end line) were 5, 8, and 10 kg, respectively.

### The concentration of OCPs in human breast milk

From the eight organochlorine pesticides analyzed in the human breast milk, only DDT and its metabolites (p,p-DDE, p,p-DDD, o,p-DDT and p,p-DDT) were detected. The mean concentration of the pesticides detected is given in milk fat weight bases. The concentration of the OCPs was calculated by taking 3.5 g of fat per 100 ml of breast milk^30^. From a total of 447 milk samples analyzed, p,p-DDE and p’p-DDT were predominantly detected (100%), followed by p,p-DDD and o,p-DDT, which were detected in 53.9%, and 42.7% of the breast milk samples, respectively. The detection of p,p-DDE and p’p-DDT in the milk samples indicates both a historical and recent use of DDT in the study area. The mean concentration of total DDT at baseline (1st month), midline (6th month), and end line (12th months) were 2.25, 1.68 and 1.32 µg/g milk fat, respectively. From the MANOVA result, the DDT metabolite residues significantly differed among breastmilk samples of the three selected stages (1st month, 6th month, and 12th months) (P < 0.0001).

The trend of the concentration of total DDT was decreasing in breast milk through the one-year lactation period (1st month > 6th month > 12th months). The Repeated Measure ANOVA result has revealed that the mean total DDT values between the three time measurement are significantly different (p = 0.002). This indicates that OCP burden were more at the time of giving birth which leads sharing of the pesticides to infants during breastfeeding. This result is supported by a study done in India, which revealed that in each lactation period there is a loss of OCPs from the mother's body involved in the nursing of their children^20^. Besides, a study was done by^28^, indicated that the concentration of DDT and its metabolites decreases through the one year lactation period. From the three sampling areas, the mean concentration of total DDT in the breast milk of mothers living in Deneba (2.14 µg/g milk fat) was greater than Serbo (1.73 µg/g lipid) and Asendabo (1.68 µg/g milk fat) districts. The concentration of total DDT in Deneba is higher than the two districts but does not have a statistically significant difference (p = 0.124) from the post hoc analysis. There is no significant difference (p = 0.816) in mean total DDT concentration between mothers living in rural and towns (urban) of the three locations. This may be due to both the urban and rural areas are malaria endemic areas and IRS is common besides agricultural use. The mean concentrations of the DDT metabolites were in the order of p’p-DDE > p’p-DDT > o’p-DDT > p’p-DDD. From the post hoc analysis, the concentration of the four DDT metabolites is significantly different from each other (p < 0.001). To differentiate between the recent or historical use of DDT in the study areas the ratio of DDT/DDE was calculated and presented in Table 1. The ratio of /DDT/DDE was less than five (< 5) for all the milk samples in the three study locations and time points. According to^20,36^, the ratio of DDT/DDE is mostly used as an indicator of the exposure history of DDT, where a high DDT/DDE ratio (> 5) represents recent exposure. In contrast, a low DDT/DDE ratio (< 5) indicates historical exposure to DDT. In the last time, the use of DDT for the control of malaria vectors as IRS was widespread because of southwest Ethiopia in general, and the three sampling locations, in particular, are highly malarious areas in Ethiopia^22,23^. According to^3^, breastfeeding women in IRS with DDT treated houses can transfer it to their infants via lactation. The mean concentration of total DDT in the study area was above the maximum residue limit (MRL) set by FAO/WHO (0.02 mg/kg milk fat)^37^. This indicates the illegal use of these environmentally persistent and highly bioaccumulative OCPs in the study areas.

**Table 1:** Summary statistics of DDT and its metabolite concentration (µg/g milk fat) in breast milk by sampling time points, location, and a lifetime of DDT application(DDT/DDE).

	p,p-DDE	p,p-DDD	o,p-DDT	p,p-DDT	∑DDT	DDT/DDE
**Timepoints**						
**Baseline (n = 166)**						
Mean	0.94	0.09	0.49	0.72	2.25	0.77
Median	0.67	0.02	0.20	0.45	1.62	
StDv	1.11	0.17	0.74	1.07	2.61	
Min	0.03	ND	ND	0.01	0.05	
Max	10.8	1.17	6.02	10.5	20.0	
**Midline (n = 144)**						
Mean	0.74	0.08	0.36	0.49	1.68	0.66
Median	0.51	0.01	0.00	0.35	1.31	
StDv	0.71	0.16	0.48	0.49	1.44	
Min	0.03	ND	ND	0.04	0.14	
Max	3.99	1.05	2.54	3.04	7.53	
**End line (n = 137)**						
Mean	0.61	0.08	0.27	0.36	1.32	0.59
Median	0.43	0.00	0.00	0.28	0.90	
StDv	0.62	0.17	0.44	0.40	1.26	
Min	0.04	ND	ND	0.01	0.06	
Max	4.90	0.97	2.21	3.37	7.02	
**Breast milk sampling locations**						
**Asendabo(n = 256)**						
Mean	0.77	0.08	0.33	0.51	1.68	0.66
Median	0.51	0.02	0.00	0.33	1.19	
StDv	0.99	0.17	0.46	0.62	1.88	
min	0.03	ND	ND	0.01	0.05	
max	10.8	1.17	2.88	5.03	16.9	
**Deneba (n = 85)**						
Mean	0.83	0.13	0.52	0.67	2.14	0.81
Median	0.69	0.03	0.46	0.38	1.53	
StDv	0.61	0.21	0.80	1.21	2.44	
min	0.09	ND	ND	0.03	0.14	
max	3.21	1.05	6.02	10.5	20.0	
**Serbo (n = 106)**						
Mean	0.75	0.05	0.42	0.51	1.73	0.68
Median	0.51	0.00	0.00	0.33	1.26	
StDv	0.72	0.10	0.64	0.54	1.65	
min	0.03	ND	ND	0.01	0.06	
max	3.99	0.51	3.55	3.04	8.90	
MRL					0.02	

ND = Not Detected, MRL = Maximum Residue Limit.

Results from Table 2, indicate the comparison of the mean concentration of total DDT in the three regions of our study areas was lesser than the previous study in southwestern Ethiopia. This may be due to the decreasing use of DDT in Ethiopia. In addition, the ratio of DDT/DDE was 3.9, 2.4 and 2.1 times lesser than the above study which also indicates historical use of DDT in three regions of Southwest Ethiopia. The mean concentration of DDT in the three locations of our study is greater than the study done inGhana, Egypt, and the Punjab state of India. This may be due to the haphazard use of this pesticide in the area or due to the three regions in our study where malaria-endemic through the use of DDT was common. The mean concentration of total DDT from Asendabo, Deneba and Serbo was 7.4 times lesser than the study in four South African regions. This may be due to the high usage of DDT in South Africa compared to Ethiopia.

**Table 2:** Comparisons of the mean concentration (µg/g milk fat) of DDT and its metabolites in three regions with literatures.

Countries	Places of study	P′P-DDT	O′P-DDT	P′P-DDE	P′P-DDD	∑DDT	DDT/DDE	References
Ethiopia	Asendabo	0.510	0.330	0.770	0.080	1.680	0.66	This study
	Deneba	0.670	0.520	0.830	0.130	2.14	0.81	
	Serbo	0.510	0.420	0.750	0.050	1.73	0.68	
Ghana	Accra	0.003		0.023	–	0.026^a^	0.13	^38^
Egypt	Assiut University	0.024	0.006	0.495	0.01	0.517	0.048	^39^
Ethiopia	Asendabo	12.20	–	4.58	0.39	17.17^b^	2.66	^23^
	Jimma	9.38	–	4.76	0.32	14.46^b^	1.97	
	Serbo	3.55	–	2.52	0.35	6.42^b^	1.41	
South Africa	Manguzi	10.0	1.70	11.00	3.50	25.00	1.1^c^	^3^
	Mseleni	4.6	0.36	13.00	1.40	17.00	0.35^c^	
	Dididi	2.30	0.670	6.80	0.60	11.00	0.34^c^	
	Gwaliweni	1.10	0.120	1.10	0.78	1.60	1.00	
India	Punjab (for Primipara)	0.085	–	1.751	0.322	2.158^b^	0.049^c^	^14^
	Punjab (for Multipara)	0.127	–	0.252	0.093	0.472^b^	0.503^c^	

^a^∑DDT = P′P-DDT + P′P-DDE, ^b^∑DDT = P′P-DDT + P′P-DDE + P′P –DDD, c.

**Figure 2:**
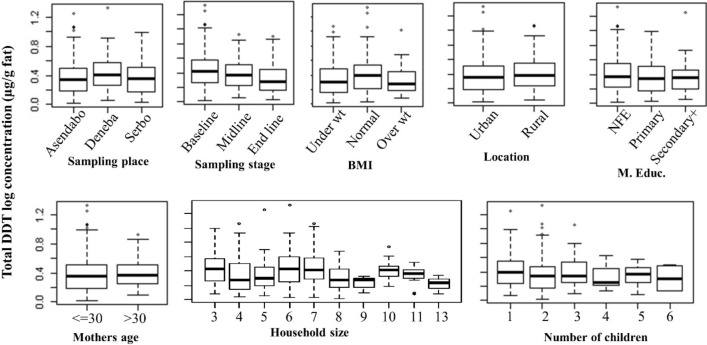
Box and whisker plot of total DDT concentration (µg/g milk fat) in relation to different categorical variables. NFE = No formal education, BMI = Body mass index, M. Educ. = educational status of mothers.

The post hoc analysis presented in a box and whisker plot (Fig. 2), indicates the concentration of total DDT among mothers in the study area in relation to the different predictor variables. Total DDT concentration was significantly different (p < 0.0001) between the milk samples collected at baseline (1st month), midline (6th month) and end line (12 months). With regards to the number of children, the concentration of total DDT was significantly decreasing in milk samples collected from mothers with more number of children (p < 0.029). Other studies on OCPs in breast milk showed that mothers breast feed their first child seems to transfer significantly higher OCPs residues compared to mothers nursing the second and third child which is in consistent with our study. The authors of these studies concluded that there is sharing of the pesticides from the mother’s body to their children^40–42^. In addition, this may be due to the fact that mothers with more number of children may not go to the agricultural fields or are not involved in pesticide spraying because they are taking care of their children at home rather primipara mothers. Hence, they may have less exposed to these pesticides. Likewise, the educational status of the breast milk donor mothers has an impact on their exposure to OCPs as most mothers in this study have no formal education (48.8%). The concentration of total DDT is decreased in mothers who have educational status of secondary school and above. This may be due to the fact that educated mothers may protect themselves from exposure by using personnel protective cloths in the field during spraying or household spraying for malaria control.

Results of multiple linear regression indicate that the age of milk donor mothers and number of children were significant predictors of the total DDT concentration in the human breast milk (p < 0.05). The body mass index (BMI) of the mothers was also a significant predictor of the contamination of breast milk by total DDT at a p < 0.1 (Table 3).

**Table 3:** Generalized linear mixed model characteristics predicting the total DDT concentration in human milk samples with demographic characters of mothers.

	Estimate	Std. Error	df	t value	Pr( >|t|)
Intercept	0.444	0.111	155.05	4.006	9.57e−05***
Mothers age	0.005	0.002	438.17	2.477	0.014*
Body mass index	− 0.007	0.004	438.02	− 1.839	0.067^.^
Number of children	− 0.029	0.011	438.04	− 2.663	0.008**
Household size	− 0.003	0.006	438.02	− 0.489	0.625
Frequency of breast feeding	0.005	0.013	438.07	0.398	0.691

Level of significance: ‘***’p < 0.001, ‘*’ p < 0.05, ‘.’ p < 0.1.

**Figure 3:**
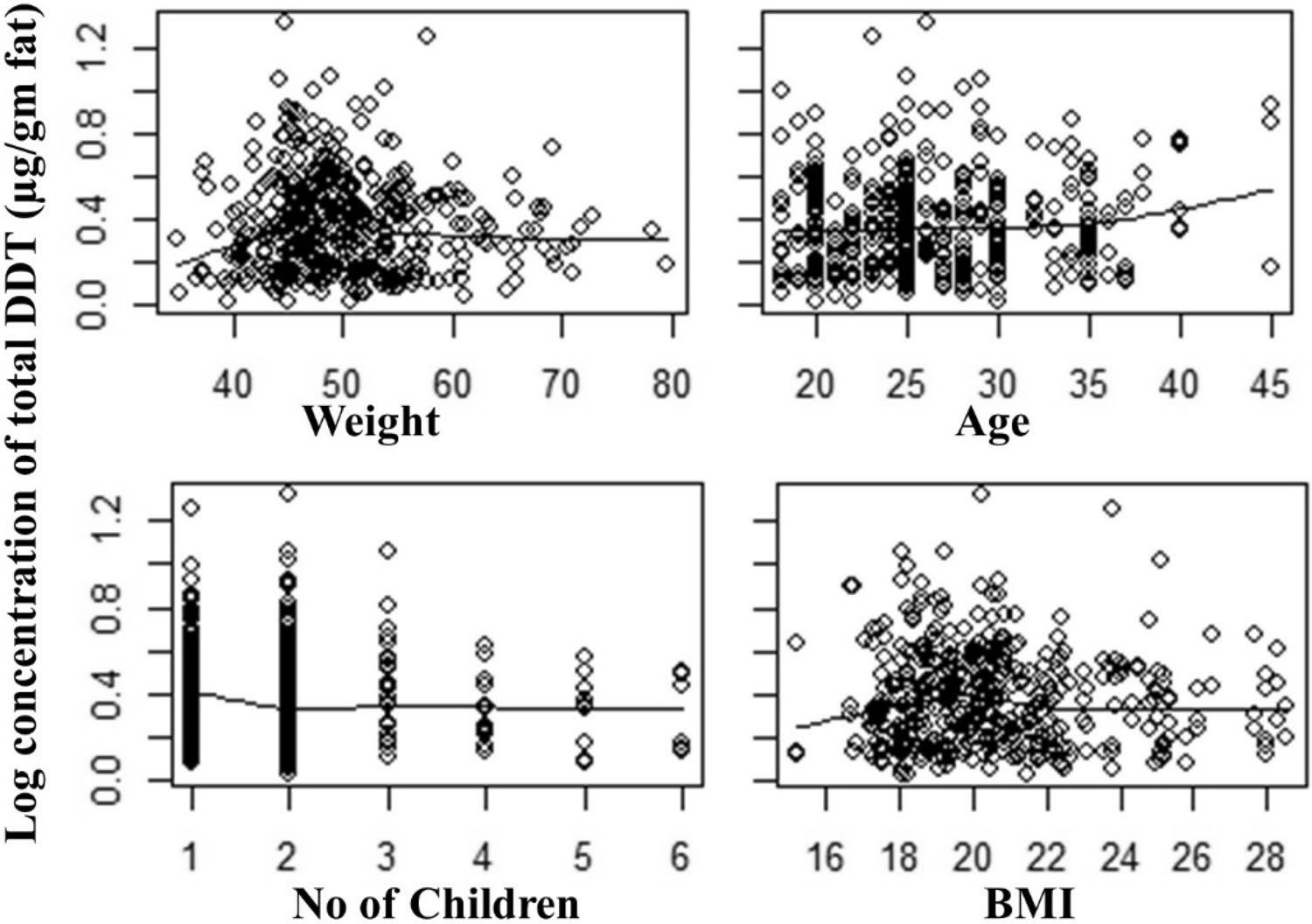
DDT related variables for breast milk samples collected in three districts of southwestern Ethiopia.

Total DDT concentrations with the number of children and body mass index are negatively correlated, while the frequency of breastfeeding and the mother's age is positively correlated. Most mothers had a body weight ranging from 40–55 kg and were found within 18–40 years. The splin regression graph demonstrated that only a few mothers had more than 25 BMI scores (Fig. 3). There is also an interesting observation that low BMI has lower levels of pesticides, which fits with the lower fat levels.

### Estimated infants daily intake

To determine the exposure of infants to these organochlorine pesticides from consumption of breast milk in the study area, the estimated daily intake (EDI) of total DDT by infants was calculated through the one-year lactation period Table 4. The EDI at 1st month > 6th month > 12 months. This may be due to the breast milk consumption is decreased as the age of the infant increases and the share of the mothers to infants through breastfeeding is decreases as the age of infants increases. The observed EDI was compared with the professional tolerable daily intake (PTDI) of total DDT in milk. According to the joint meeting of FAO/WHO guidelines, the PTDI of DDT in milk is 10 µg/kg body weight^43^.

**Table 4:** Estimated daily intake of infants (µg/kg BW/day).

Time Points of milk sample collection	Breast milk intake (g/d)	mean conc. (µg/g lipid)	EDI (µg/kg BW/day)	PTDI (µg/kg BW/day)
1st month	713.79	2.25	11.24	10
6th month	775.59	1.68	5.70	
12th month	547.96	1.32	2.53	
Average	2037.34	5.25	19.47	

From the present study results, the estimated daily intake (EDI) of infants' first month lactation period is 11.24 µg/kg BW Day-1. In this study, the EDI of infants from breast milk consumption during the first month breastfeeding period is greater than the PTDI probably due to the high milk intake at 1st month. The EDI higher than the PTDI indicates that there might be a health risk for infants at an early stage in the study areas. While extended exposure of infants to OCPs can lead to endocrine and reproductive disorders, such as gonadal changes, changes in reproductive behavior, thyroid dysfunction, organ malformations, and many others^44^. For the 6th and 12th month lactation period the EDI is less than the PTDI which indicates as the age of the infant increases consumption of breast milk also decreases and indicates less health riskfor infants. Our result is far less than the mean estimated daily intake obtained earlier from a study done in southwest Ethiopia^23^. This may be due to the concentration of DDT decreases in the environment from time to time. Its application for malaria control and agricultural uses is decreased in the study areas and Ethiopia in general.

### Limitation of the study

Although OCPs are persistent, the milk samples were stored at − 80 °C for five years before analysis was done, as the milk samples were primarily collected for other study^26,27^.

## Conclusions

Our study investigated the level of organochlorine pesticides (OCPs) in human breast milk collected from the three districts of Jimma zone southwestern Ethiopia. It also has determined the risk of infants' exposure to OCPs. The study revealed that DDT and its metabolites were detected, while Aldrin, Dieldrin, Endosulfan α, and Hexachlorobenzene were not detected. The ratio of DDT/DDE for the three regions was < 5 has indicated that large-scale historical DDT uses in southwestern Ethiopia would pose a similar health risk with the parent compound DDT. The mean concentrations of total DDT that infants are getting through breastfeeding were above the MRL set by FAO/WHO. It is also noted that as the number of children for a mother increases the residue of total DDT decreases. This may be due to the sharing of the residues during the nursing of the consecutive children. Most mothers have no formal education and the residue of DDT in the breast milk of mothers with an educational level of secondary and above were less. The estimated daily intake (EDI) of infants in the present study was above provisional tolerable daily intake (PTDI) during the first month of breastfeeding which indicates that there is a health risk for infants consuming breast milk at an early stage of breastfeeding in the study areas. Therefore, yearly monitoring of these and other pesticides in human breast milk is an essential and strict follow-up of the non-use of banned pesticides in Ethiopia is mandatory.

## Data Availability

Data will be available upon request.
